# *Lavandula angustifolia* Essential Oil and Linalool Counteract Social Aversion Induced by Social Defeat

**DOI:** 10.3390/molecules23102694

**Published:** 2018-10-19

**Authors:** Lucia Caputo, Marina D. Reguilon, José Mińarro, Vincenzo De Feo, Marta Rodriguez-Arias

**Affiliations:** 1 Department of Pharmacy, University of Salerno, Via Giovanni Paolo II, 132, 84084 Salerno, Italy; lcaputo@unisa.it; 2 Departament of Psicobiology, University of Valencia, Avda. Blasco Ibáñez, 21, 46010 Valencia, Spain; reguilonmarina@gmail.com (M.D.R.); jose.minarro@uv.es (J.M.); Marta.Rodriguez@uv.es (M.R.-A.)

**Keywords:** *Lavandula angustifolia*, essential oil, linalool, social defeat

## Abstract

Many vegetable extracts, essential oils, and their main constituents are active on the Central Nervous System (CNS). In fact, they have been used as sedatives, hypnotics, or tranquilizers for their activity in treating CNS disorders. In this research, we studied the possible activities of *Lavandula angustifolia* (LA) essential oil and of its main constituent, linalool, as anti-stress compounds on anxiety and social interaction and their in vitro effects on proteins (pERK and PKA) involved in the transmission of the signal. An acute intraperitoneal injection of linalool (100 mg/kg) and of LA essential oil (200 mg/kg) reduced motor activity without any anxiolytic effect, but significantly increased social interaction. Stressed mice, after being exposed to a social defeat encounter, showed heightened anxiety and social avoidance. Acute administration of LA essential oil blocked stress-induced anxiety, while linalool showed no effects. However, both compounds were capable of reversing social aversion, acting as antidepressant agents. Our results showed that linalool inhibits pERK and PKA expression in the SH-SY5Y cell, but no effect was detected with the LA essential oil. Therefore, the LA essential oil and linalool may be considered as useful alternative tools to the available traditional treatments for social stress-induced mental illnesses.

## 1. Introduction

Essential oils are natural complex mixtures of volatile compounds considered as multifunctional agents. Among their properties, they stimulate human smell since the olfactory information reaches a number of cortical areas without being relayed in the thalamus [1]. Aromatherapy is a complementary medical practice involving the therapeutic use of essential oils to treat various physical or psychological conditions [2]. Different studies have been carried out to evaluate the effects of essential oils and aromatic species on the Central Nervous System (CNS), reporting effects on learning, memory, and attention. Essential oils have also been proposed for the treatment of stress [3], which is one of the most prevalent psychological disorders in developed countries, leading to other clinical features, such as anxiety, insomnia, or depression. Anxiety is among the most common forms of psychopathology worldwide, characterized by shortness of breath, heart palpitations or pale skin. Its prevalence as a medical condition is increased in recent years, since humans are continually exposed to various anxiety-promoting situations in their surrounding environment [4]. Usually, the treatment for persistent anxiety requires the use of benzodiazepines, but they have many side-effects and they are also involved in withdrawal and “rebound effects” as a result of discontinuing their administration. Therefore, the alternative management of anxiety and other social consequences of stress is of great importance in contemporary urban life [5,6].

*Lavandula angustifolia* (LA) Mill. (lavender, Lamiaceae) is an aromatic plant used in folk medicine for the relief of stress and anxiety [7] and can be considered one of the best-selling over-the-counter herbal remedies. The genus Lavandula includes more than 30 species and it is widely distributed in the lands surrounding Mediterranean Sea and in the archipelagoes of the Atlantic Ocean [7]. Lavender aromatherapy has been proven to reduce preoperative anxiety [8] or anxiety in postmenopausal women [9]. In a recent study, lavender oil was found to be more potent than ibuprofen in alleviating stress related disorders in rats subjected to restraint stress [10]. LA represses the principal pro-inflammatory cytokines and their receptors, by exerting an anti-inflammatory and immune regulatory role [11]. Moreover, this essential oil improves scopolamine-induced cognitive deficits in mice, by exerting a neuroprotective effect in this Alzheimer disease model [12]. This medicinal plant has been approved by several international organizations (World Health Organization, the European Scientific Cooperative on Phytotherapy or the European Medicines Agency) as a remedy to relieve stress, restlessness, and anxiety [13]. Specifically, the European Medicines Agency recommends a daily oral dose of LA from 20 to 80 mg to relieve mild symptoms of mental stress and to insomnia. A recent report has shown that LA exerts affinity for several specific receptors on the CNS [14].

Linalool is a monoterpene reported to be a major component of various aromatic plant essential oils, including LA [15]. In early studies, Elisabetsky and coworkers showed that this compound possesses dose-dependent sedative effects in the rat cerebral cortex [15,16]. Moreover, linalool exhibits diverse pharmacological activities, including antioxidant, anti-inflammatory, and cardiovascular effects in hypertensive rats [17,18,19]. A recent study suggested that chronic linalool administration induces a reduction of memory loss and emotional impairments in a mouse model of Alzheimer’s disease [20]. Linalool also shows antidepressant-like effects, decreasing immobility time in the tail suspension test [21]. In our previous studies, we reported that *Lavandula angustifolia*, *Coriandrum sativum*, and *Laurus nobilis* essential oils and their main components, i.e., linalool and 1,8-cineole, influenced adenylate cyclase 1 (ADCY1) expression [22,23]. ADCY1 possesses a crucial role in the CNS; in fact, synaptic plasticity and memory formation depend on optimal cAMP levels. Adenylyl cyclase activates several signal transduction pathways, including that of Erk/MAPK and PKA [24].

One of the most recognized models of social stress in animal models is the resident-intruder paradigm, which induces anxiety and depressive-like behaviors in the intruder mice [25]. Numerous studies have reported long-lasting anxiety, anhedonia, and depressive-like symptomatology in defeated animals, which also show increased vulnerability to mental illnesses and higher drug intake [26,27,28,29,30]. Although, treatment with essential oils in humans reduces anxiety and depressive symptoms [31], the effect of these essences has not been directly studied in socially stressed animals. However, preliminary studies in humans under social stress indicate a potential positive effect [32]. In this line, a series of recent reports observed that another essential oil (*Ocimum basilicum*) reduces memory impairment, hippocampal neurodegeneration, and depressive-like symptomatology caused by the chronic unpredictable stress model in mice [33,34].

Based on the above-mentioned results, the aim of this study was to evaluate the in vitro and in vivo effects of LA essential oil and its main constituent linalool on the anxiety and avoidance levels of socially stressed mice. At first, the acute and chronic effects of both compounds using a model of social stress, the social defeat paradigm, were evaluated. Repeated environmental stress induces behavioral changes, such as depression and heightened anxiety in various animal species [35,36]. Since social defeat stress induces decreased sucrose preference, social avoidance and heightened anxiety in mice ameliorated by an antidepressant treatment, it has been proposed as a mouse model of depression. The growing awareness of the adverse effects of central nervous system drugs has led to the development of new strategies and the search for safer pharmacological agents in mental health. Furthermore, in order to clarify their possible subcellular mechanism, we evaluated if LA essential oil and linalool can influence pERK and PKA expression in SH-SY5Y cells.

## 2. Results

### 2.1. Effect of LA Essential Oil and Linalool on Anxiety and Social Behaviors

#### 2.1.1. Experiment 1: Acute Effects of Linalool and LA Essential Oil

##### Open Field

**Figure 1:**
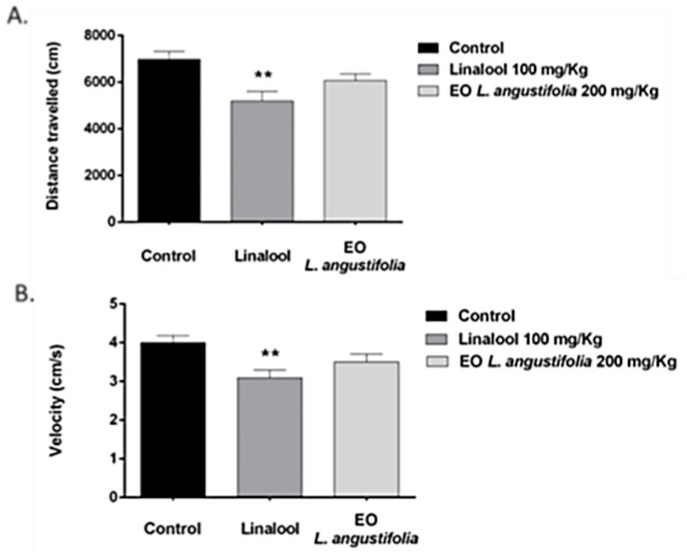
Effects of linalool (100 mg/kg) and *Lavandula angustifolia* (LA) essential oil (200 mg/kg) in the open field test. (**A**) Effects on the distance travelled (cm) by mice; (**B**) Effects on the velocity (cm/s) of mice. The bars represent the mean ±SEM of animals in the different treatment groups. ** *p* < 0.01 significant difference compared to control.

The results of the acute effect of LA and linalool in the open field test are shown in Figure 1. As indicated, the administration of 100 mg/kg of linalool had a significant effect on the distance travelled [F (2,42) = 6.736; *p* < 0.01] and the velocity [F (2,42) = 5.427; *p* < 0.01]. Mice treated with linalool travelled less distance with lower velocity compared to mice treated with LA essential oil and the control group (*p* < 0.01). Analyses of the time spent in the center of the open field and the frequency of entries in the central area of the open field did not reveal any significant effect (data not shown).

##### Elevated Plus Maze

**Figure 2:**
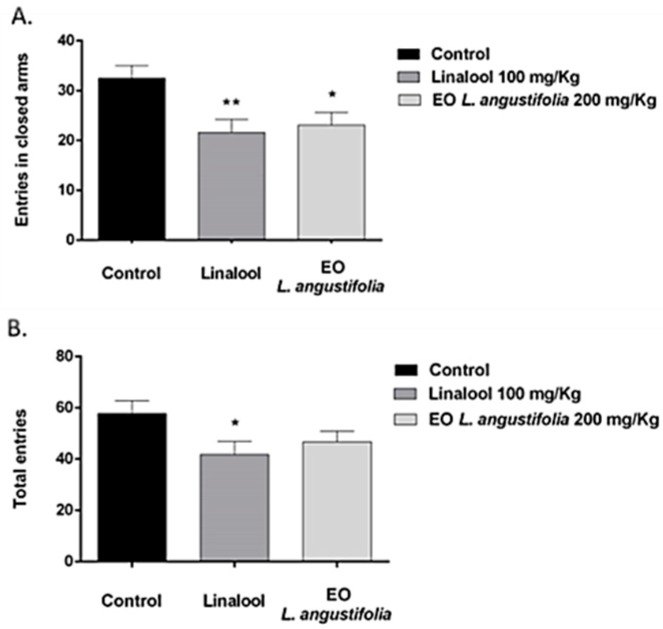
Effects of linalool and LA essential oil acute administration in the elevated plus maze (EPM) test. Animals were divided into the following three treatment groups: Control (saline) (*n* = 15), Linalool (*n* = 15) and LA essential oil (*n* = 15). Results are presented as mean values ±SEM. (**A**) Entries in closed arms. * *p* < 0.05; ** *p* < 0.01 differences with the control group. (**B**) Total entries. * *p* < 0.05 difference with the control group.

The elevated plus maze (EPM) data (Figure 2) revealed that the number of entries in the closed arms was lower in animals treated with linalool and LA essential oil in comparison to the controls (*p* < 0.01 and *p* < 0.05, respectively) [F (2,42) = 5.437; *p* < 0.01]. In addition, the number of total entries [F (2,42) = 2.964; *p* > 0.05] was lower in mice treated with 100 mg/kg of linalool than in the control group.

##### Social Interaction Test

Table 1 presents the behavior data collected in the social interaction test. The results concerning the time spent in Social Investigation [F (2,42) = 5.615; *p* < 0.05] and the mean time spent in each social contact (Unit of Social Investigation) [F (2,42) = 21.43; *p* < 0.0001] showed that mice receiving linalool or LA essential oil increased the time spent in this behavior in comparison to the saline control group (*p* < 0.05 for time in social investigation; *p* < 0.0001 and *p* < 0.01, for unit of social investigation, respectively). On the other hand, mice treated with linalool or LA essential oil spent less time in Non-Social Exploration compared to controls (*p* < 0.01).

#### 2.1.2. Experiment 2: Linalool and LA Essential Oil Effects When Administered after Social Stress

##### Elevated Plus Maze

The EPM data (Table 2) revealed an effect of treatment on the time [F (3,41) = 4.572; *p* < 0.01] and the percentage of time [F (3,41) = 3.19; *p* < 0.03] spent in the open arms of the maze. Defeated mice spent less time in the open arms of the maze than the control non-stressed animals (*p* < 0.05). Defeated mice treated with linalool showed a decrease in the time (*p* < 0.05) and the percentage of time (*p* < 0.02) spent in the open arms.

The number of entries in the closed [F (3,41) = 6.135; *p*< 0.001] and open [F (3,41) = 4.306; *p* < 0.05] arms were also lower for defeated animals treated with linalool compared to the non-stressed group (*p* < 0.001 and *p* < 0.05, respectively). Moreover, defeated mice treated with linalool or LA essential oil spent more time in the closed arms than those in the saline exploration control group (*p* < 0.01 and *p* < 0.05, respectively).

##### Social Interaction Test

Data for the different behavioral parameters evaluated in the social interaction test are presented in Table 3. Defeated mice decreased the time spent in Social Investigation [F (3,41) = 6.467; *p* < 0.01] in comparison to the exploration control group (*p* < 0.01). However, defeated mice treated with linalool or LA essential oil spent the same time in Social Investigation than non-stressed controls (*p* < 0.01). Accordingly, defeated mice treated with saline spent more time in Non-Social Exploration [F (3,41) = 4.579; *p* < 0.01] in comparison with the control group (*p* < 0.05) and those defeated but treated with linalool (*p* < 0.01) or LA essential oil (*p* < 0.05).

#### 2.1.3. Experiment 3: Effects of Acute or Chronic Administration of Linalool and LA Essential Oil before Social Defeat in Mice

##### Social Defeat Encounters

The ANOVA for the behaviors of the resident mice (see Table 4) showed less attack behavior [F (4,55) = 13.08; *p* < 0.0001] against mice treated with a single dose of linalool (*p* < 0.01) and with an acute or chronic administration of LA essential oil (*p* < 0.001). They also took longer time to perform the first attack [F (4,55) = 3.379; *p*< 0.05] to animals treated with chronic *L. angustifolia* EO (*p*< 0.05 with respect Control group). Resident mice also displayed higher threat behavior [F (4,55) = 123.7; *p* < 0.001] against mice treated with a chronic administration of LA essential oil.

Intruder mice treated with a chronic administration of LA essential oil displayed less defensive/submissive [F (4,55) = 3.523; *p* < 0.05] and avoidance [F (4,55) = 3.302; *p* < 0.05] behaviors than the controls (Table 4). Moreover, intruder mice treated with a single dose of *L. angustifolia* took more time to perform their first defensive/submissive behavior [F (4,55) = 4.195; *p* < 0.05] than the controls.

##### Elevated Plus Maze

The ANOVA (Table 5) revealed an effect of treatment on the time [F (5,63) = 5.086; *p* < 0.001] and the percentage of time [F (5,63) = 2.736; *p* < 0.02] spent in the open arms of the maze. Defeated mice treated with saline spent less time in the open arms of the maze than the control non-stressed animals (*p* < 0.001). In addition, defeated animals treated with acute or chronic administration of linalool also showed a decrease in the time (*p* < 0.05 and *p* < 0.01, respectively) spent in the open arms with respect to the control non-stressed mice.

Acute administration of *L. angustifolia* EO prior to SD counteracted the anxyogenic effect, although the protective effect disappeared after chronic administration (*p* < 0.03 with respect to controls in the time spent in the open arms). Moreover, defeated mice treated with a chronic administration of *L. angustifolia* essential oil spent more time in the central platform [F (5,84) = 2.313; *p* > 0.05] than the control non-stressed animals (*p* < 0.05). The ANOVA of EPM data revealed that the number of entries in the closed arms [F (5,84) = 1.791; *p* > 0.05], and the number of total entries [F (5,84) = 1.822; *p* > 0.05] were lower in animals treated with chronic administration of Linalool before social defeat than control non-stressed animals (*p* < 0.05).

##### Social Interaction Test

Table 6 reports the different behavioral parameters evaluated in the social interaction test. SD induced a decrease in Social Investigation [F (5,66) = 5.091; *p* < 0.001)] with more time spent in Non-Social Exploration [F (5,66) = 4.673; *p* < 0.01] in comparison to the exploration control group (*p* < 0.01 in both cases). Moreover, mice treated with an acute or chronic administration of linalool or with a chronic administration of *L. angustifolia* essential oil before SD did not show this effect (*p* < 0.001 and *p* < 0.01 for acute and chronic linalool; *p* < 0.01 for chronic *L. angustifolia* essential oil with respect to the SD saline group). Only those mice chronically treated with Linalool [F (5,66) = 3.511; *p* < 0.05] showed longer latency to perform the first social contact (*p* < 0.05 with respect to the Controls and SD saline groups).

### 2.2. Western Blot Analysis: pERK and PKA

**Figure 3:**
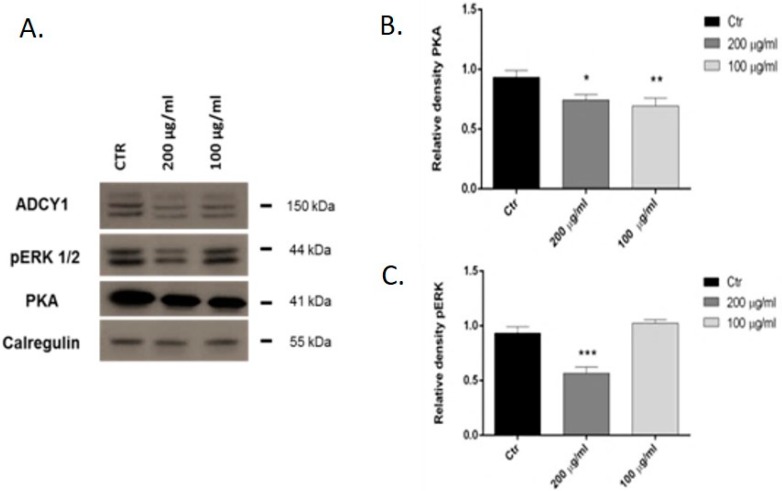
Representative Western blot (**A**) and relative expression levels of the PKA (**B**) and pERK (**C**) in SH-SY5Y cells treated with linalool. Values are the mean ± SD in each group (*n* = 3). * *p* < 0.05, ** *p* < 0.01, *** *p* < 0.001, compared to the control (ANOVA followed by Dunnett’s multiple comparison test).

We investigated the effects of *L. angustifolia* essential oil and its main constituent, linalool, in the human neuroblastoma SH-SY5Y cell line. Treatments with 200 μg/mL of linalool for 24 h significantly inhibited pERK expression and concentrations of 200 and 100 μg/mL reduced PKA expression. A more representative Western blot and quantitative densitometric analysis for pERK and PKA protein expressions is shown in Figure 3. *L. angustifolia* essential oil had no significant effects on pERK protein and PKA expression (data not shown).

## 3. Discussion

Our study confirms that essential oils, such as *L. angustifolia* EO, and its main constituent, linalool, could be a useful tool to treat anxiety and social disorders induced by social stress. In acute doses, both compounds showed sedative effects, with the reduction in motor activity being more intense after linalool administration. Although, no anxiolytic effect was detected in non-stressed mice, both compounds increased social interaction behaviors. More interestingly, this increase is also observed in socially stressed mice treated with LA essential oil or linalool. Administered after or before an episode of social defeat, acute or chronic administration of linalool and LA essential oil are capable of reversing social aversion, thereby acting as antidepressant agents. At the doses of 100–200 μg/mL, linalool also inhibited pERK and PKA expression whereas LA essential oil (100–400 μg/mL) showed no significant effects on the expression of these proteins in SH-SY5Y cells.

To test the behavioral effects of these natural substances, we used three different conditions: (i) acute administration without stress, to evaluate basal effects; (ii) acute administration after a single episode of social stress; and (iii) acute or chronic administration before social stress. Under basal conditions, linalool impaired motor activity in OF1 mice. In the open field test, mice treated with linalool traveled a shorter distance with less velocity than their respective control group. In agreement with these results, Shaw and coworkers demonstrated that rats treated with LA essential oil show reduced peripheral movement [37]. Although in our experiment we did not observe impairment in motor activity after LA essential oil administration, the different methods of administration (inhalation vs. ip injection) and the doses employed can explain these differences.

On the other hand, neither of the compounds exert any effect on the anxiety profile, although the results of the EPM for linalool treated mice are in agreement with the impairment of motor activity. The number of entries in the closed arms was lower in animals treated with linalool in comparison to the control group. We can find contradictory reports in the literature on LA essential oil or linalool with respect to their effects on the EPM. Chioca and collaborators observed that mice exposed to inhaled lavender essential oil increased the number of entries and time spent in the open arms [38], although Kumar and coworkers reported that Silexan (a standardized essential oil produced from LA flowers with 36% linalool and 34% linalyl acetate) decreased the number of closed arm entries in the elevated plus maze [39]. These discrepancies are also observed in the studies performed with linalool, as Linck and coworkers demonstrated that linalool reduced locomotor activity in mice, although Coelho and coworkers showed that it reduced the immobility time in the tail suspension test [21,40].

In contrast to the lack of anxiolytic effect on the EPM, mice treated with linalool or LA essential oil spent more time in social investigation during the social interaction test when compared to the control group. This result agrees with that of Linck and coworkers, which revealed a significant effect of inhaled linalool in increasing the social interaction levels [5].

As previously reported, an episode of social defeat induced an anxiolytic response with a decrease in the time spent by defeated mice in the open arms of the EPM [41,42]. In addition, we also observed that social defeat induced social avoidance, once more in agreement with previous reports [42,43]. Defeated mice spent less time in social contacts, increasing non-social exploration behavior in comparison with non-stressed controls.

The administration of LA essential oil or linalool (Table 3) after the social defeat episode is capable of partially counteracting the increased anxiety induced by social stress. Although stressed mice treated with LA essential oil spent less time in the open arms than controls, this decrease was not statistically significant. This effect (less time in open arms) was significant after linalool administration. However, both essential oils were capable of counteracting the social avoidance induced by social defeat. In fact, both linalool and LA essential oil increased the time spent in social interactions with respect to the SD saline group. There are no previous reports evaluating the action of essential oils in social defeat effects. In our third study, LA essential oil or linalool were administered prior to the social defeat episode, therefore it was necessary to evaluate their effect during the social defeat encounter. Firstly, our ethological evaluation revealed that resident mice delayed to attack the intruder treated with a chronic LA essential oil administration and when they did, they attacked with less intensity. This decrease in aggression was partially observed in attacks against mice acutely treated with linalool, that were less frequently attacked by the resident mice. The fact that the decrease in aggression by resident mice was primarily observed when it was against mice acutely treated with LA or linalool suggests that some kind of tolerance is developed in mice chronically treated with these essential oils. Intruder mice receiving chronic LA administration also showed less submissive and defensive behaviors, probably due to their less intense experience of aggression. We hypothesized that since the propensity of odorants and pheromones in urine is known to increase male mouse aggressive behavior [44,45], LA essential oil or acute linalool (in a lesser extend) administration can hide or change the perception of these pheromones and therefore decrease the aggressive response from the resident mouse. However, it is important to notice that all the intruder animals, irrespective of their treatment group were actually defeated, and therefore experienced social defeat.

Similar to the effects observed with the administration of LA essential oil after a social defeat, an acute administration of LA prior to a social defeat blocked the increased anxiety response induced by this stress. The fact that chronic LA failed to induce the same results suggest once more the development of tolerance to LA use. In agreement with the previous results, linalool did not show any effect on the anxiety profile. Therefore, we suggest that another compound present in the LA essential oil different from linalool could be responsible for counteracting the social stress-induced anxiety. In LA essential oil, 59 compounds have been identified, accounting for 97.3% of the total oil. Linalool (33.1%), linalyl acetate (10.4%), 1,8-cineole (8.0%) and borneol (4.5%) are the main components [22].

On the other hand, as we have previously observed, when administered after social stress, previous acute or chronic administration of LA essential oil and linalool blocked social avoidance-induced by social defeat. Only one previous report showed a stress relief effect of LA aroma on stress markers in humans exposed to an arithmetical task [32]. No studies have been performed in animal models representing social stress, although the anti-stress properties of these compounds have been demonstrated after restraint stress [10]. Another essential oil (*Ocimum basilicum*) reduces depressive-like symptomatology caused by chronic unpredictable stress model in mice [33]. Based on the above-mentioned effects of LA and linalool during the social defeat encounter, a specific pro-social action based on the change of odor recognition cannot be ruled out. Olfactory signals can be segregated into associative and specialized odors. Associative odors do not inherently encode behavioral meaning, but specialized odors activate neural pathways that are pre-set with meaning. Specialized odors are thought to include pheromones [46]. Pheromones are molecules produced by one individual that act as ‘ectohormones’ to hijack behavior when detected by another member of the same species [47]. The biochemical properties of these ligands are diverse (from proteins to peptides or small organic volatiles) as are the molecular identities of their cognate sensory neurons [48]. Normal odors can appear to act as pheromones when coupled with experience, by similarly evoking a range of social behaviors with emotional valence, such as fear and attraction [49].

Finally, we tested the ability of these compounds on the expression of pERK and PKA in SH-SY5Y cells. The ability of PKA and Rap1 to couple to ERK activation has significant implications for neuronal signaling [50]. In fact, PKA stimulation of ERK activity may regulate both neuronal survival and synaptic plasticity [51,52]. Our results showed that linalool inhibits pERK and PKA expression in SH-SY5Y cell. The inhibition of these proteins, and of ADCY1 and ERK expression, as shown in our previous studies [22], could explain the dose-dependent sedative effects in the CNS described by Elisabetsky and coworkers [15,16]. Furthermore, given that high levels of ERK activation correlated with the occurrence of both allodynia and hyperalgesia in several pain models, it is likely that this mechanism contributes to the plastic neuronal changes associated with chronic pain. On these grounds, inhibitors of ERK phosphorylation could be used to reverse altered pain states [53].

LA essential oil showed different effects, and in fact had no influence on pERK and PKA expression. Moreover, in our previous study we showed that there is a different effect of this essential oil with respect to its principal component, linalool, on ADCY1 expression [22]. Most likely, this essential oil influenced other intracellular pathways to determine a concentration-dependent inhibition of neuronal networks and provoke the sedative effect on the CNS described in other studies [15,16]. Recent reports show that LA essential oil lacked appreciable affinity for norepinephrine or dopamine reuptake transporters, as well as monoamine oxidase-A or gamma-aminobutyric acid-A receptors, although LA essential oil and its main components exert affinity for the glutamate NMDA-receptor in a dose-dependent manner and also bind to the serotonin transporter [14,31]. These actions have been confirmed using positron emission tomography and magnetic resonance imaging scanning after 8 weeks of administering LA essential oil, since a reduced binding potential at the 5HT1A receptor in the hippocampus and the anterior cingulate cortex has been observed [54,55].

The well-known side effects of common antidepressant drugs, such as benzodiazepines and selective serotonin reuptake inhibitors, have led to an increase in the use of natural therapies. Our results confirmed that LA essential oil exhibited an anti-stress effect, decreased anxiety, and social avoidance without intense sedation, which is advantageous compared with the present treatments. In addition, LA lacks a withdrawal syndrome and is not thought to have abuse potential [56]. Its main constituent, linalool, did not decrease anxiety, but it showed an intense prosocial effect.

Available trials support the short-term efficacy of the standardized lavender oil extract in the treatment of anxiety disorders [31], but many questions remain unanswered regarding their use. Essential oils are able to traverse cell membranes and exhibit pharmacologic effects, making them drug-like and increasing suitability for potential pharmaceutical applications [57].

## 4. Materials and Methods

### 4.1. Animals

A total of 70 male mice of the OF1 strain were purchased from Charles River (Barcelona, Spain) at 42 days of age. They were housed in groups of four in plastic cages (25 × 25 × 14.5 cm) for 8 days prior to the initiation of experiments, under the following conditions: constant temperature (21 ± 2 °C), a reversed light schedule (white lights on: 19:30–07:30), and food and water available ad libitum, except during behavioral tests. All procedures were conducted in compliance with the guidelines of the European Council Directive 2010/63/UE regulating animal research and were approved by the local Ethics Committee for Experimentation and Animal Welfare of the University of Valencia.

### 4.2. Drug Treatment

Animals were injected intraperitoneally with 100 mg/kg of linalool (Sigma Aldrich, St. Louis, MO, USA) or 200 mg/kg of *L. angustifolia* essential oil (linalool 33.1%) (obtained and analyzed in our previous work [22]). Linalool and essential oil were solubilized in physiological saline solution with 2% Tween-80 and 1% DMSO. The control groups were injected with physiological saline (NaCl 0.9%), which was also used to dissolve the drug. The doses of linalool and *L. angustifolia* essential oil used to test the effects on behavior of mice in different situations were selected on the basis of previous studies [12,21,58].

### 4.3. Experimental Design

The first experiment was carried out in non-stressed mice. Thirty minutes after the administration of the linalool or LA essential oil, OF1 mice animals performed the Open Field, the Elevated Plus Maze (EPM) and the Social interaction test. Three groups were employed in this experiment: control (*n* = 15), linalool (*n* = 15), and *L. angustifolia* EO (*n* = 15).

In the second experiment, the same OF1 mice (40 days after experiment 1) were exposed to the SD stress condition followed by an intraperitoneal injection of linalool or LA essential oil 10 min later and performing the EPM and Social interaction tests 30 min later. Four groups were employed in this experiment: Control (Explora) (*n* = 11), SD Saline (*n* = 10), SD linalool (*n* = 12), SD *L. angustifolia* EO (*n* = 12).

Seventeen days after the end of experiment 2, OF1 mice received an acute (a single administration the test day) or a chronic administration (daily treatment for a period of 10 days before tests) of linalool or LA essential oil. Mice were exposed to SD 30 min after the administration. Forty minutes after social defeat, they performed the EPM and Social interaction tests. The groups employed in these experiments are: Control (Exploration) (*n* = 12), SD saline (*n* = 12), SD Acute linalool (*n* = 12), SD Acute LA (*n* = 12), and SD Chronic linalool (*n* = 12), SD Chronic LA (*n* = 12).

### 4.4. Open-Field Test

Mice were habituated to an open field (30.5 × 29 × 35 cm) dark cage and were allowed to explore freely for an hour. Activity was tracked and analyzed using the EthoVision XT software (Noldus Information Technology, Wageningen, The Netherlands, http://www.noldus.com) to determine the total distance covered, the speed and the percentage of time spent in the center of the cage.

### 4.5. Elevated Plus Maze-EPM

EPM test was carried out essentially following the procedure described by Daza–Losada and co-workers [59]. The maze consisted of two open arms (30 × 5 × 0.25 cm) and two enclosed arms (30 × 5 × 15 cm), and the junction of the four arms formed a central platform (5 × 5 cm). The floor of the maze was made of black Plexiglas and the walls of the enclosed arms were made of clear Plexiglas. The open arms had a small edge (0.25 cm) to provide the animals with additional grip. The entire apparatus was elevated 45 cm above floor level. In order to facilitate adaptation, mice were transported to the dimly-lit laboratory 1 h prior to testing. At the beginning of each trial, subjects were placed on the central platform so that they were facing an open arm and were allowed to explore for 5 min. The maze was thoroughly cleaned with a damp cloth after each trial. The measurements recorded during the test period were the number of entries and the time and percentage of time spent in each section of the apparatus (open arms, closed arms, and central platform). An arm was considered to have been visited when the animal placed all four paws on it. The percentage of time spent in the open arms and the number of open arm entries are generally used to characterize the anxiolytic effects of drugs. In addition, the number of closed and total entries indicates motor activity.

### 4.6. Social Encounters

This test consisted of confronting an experimental animal with a standard opponent toward a neutral cage (61 × 30.5 × 36 cm) for 10 min following a 1-min adaptation period. Standard opponents were rendered temporarily anosmic by intranasal lavage with a 4% zinc sulfate solution 1 day before testing [60]. This kind of mouse induces an attack reaction in its opponent but does not outwardly provoke or defend itself, since it cannot perceive a pheromone that is present in the urine of the experimental animals and functions as a cue for eliciting aggressive behavior in mice with a normal sense of smell [61]. A more detailed description of the behaviors evaluated can be found in Rodríguez-Arias et al. [62].

### 4.7. Procedure of Social Defeat

The Social Defeat procedure consisted of three phases, which began by introducing the “intruder” (the experimental animal) into the home cage of the “resident” (the aggressive opponent) 10 min [63]. During this initial phase, the intruder was protected from attack, but the wire mesh walls of the cage allowed for social interactions and species-typical threats from the male aggressive resident, thus allowing for instigation and provocation [64]. The wire mesh was then removed from the cage to allow confrontation between the two animals for a 5-min period. In the third phase, the wire mesh was returned to the cage to separate the two animals once again for another 10 min to allow for social threats by the resident. Intruder mice were exposed to a different aggressor mouse during each episode of social defeat. The criterion used to define an animal as defeated was the adoption of a specific posture signifying defeat, characterized by an upright submissive position, limp forepaws, upwardly angled head, and retracted ears [62,65]. All agonistic encounters were videotaped to confirm social defeat in our experimental mice.

### 4.8. Human Neuroblastoma Cell Cultures

Human neuroblastoma (SH-SY5Y) cancer cells were cultured in RPMI medium supplemented with 1% l-glutamine, 10% heat-inactivated fetal bovine serum (FBS), 1% penicillin/streptomycin (all from Sigma Aldrich, St. Louis, MO, USA) at 37 °C in an atmosphere of 95% O_2_ and 5% CO_2_.

### 4.9. Extraction of Proteins and Western Blotting

Cells were treated with different concentrations of linalool (100–200 μg/mL) and *L. angustifolia* essential oil (100–400 μg/mL). The cells were collected after 24 h and lysed using the Laemmli buffer to extract total proteins. For Western Blot analysis, an aliquot of total protein was run on 10% SDS-PAGE gel and transferred to nitrocellulose. Nitrocellulose blots were blocked with 10% non-fat dry milk in Tris buffer saline 0.1% Tween-20 over night at 4 °C and incubated with primary anti-pERK and anti-PKA (Santa Cruz Biotechnology, Santa Cruz, CA, USA) for 3 h at room temperature. Immunoreactivity was detected through a sequential incubation with horseradish peroxidase-conjugated secondary antibody (Amersham Biosciences, Pittsburgh, PA, USA) and enhanced chemiluminescence reagents (ImmunoCruz, Santa Cruz Biotechnology, Santa Cruz, CA, USA) [66]. The density of each band was measured by using ImageJ software (WS Rasband, Image J, NIH, Bethesda, MD, USA).

### 4.10. Statistical Analyses

Data for the behaviors evaluated by the open field, the EPM, and social interaction test as well as for the data from the resident–intruder test were analyzed using a one-way ANOVA with one between variable: treatment (with three levels in experiment 1 and 2 or six in experiment 3). All experiments in vitro were carried out in triplicate. The data for each experiment were statistically analyzed using the GraphPad Prism 6.0 software (GraphPad Software Inc., San Diego, CA, USA) followed by a comparison of means (two-way ANOVA) using Dunnett’s multiple comparisons test, at a significance level of * *p* < 0.05.

## Figures and Tables

**Table 1:** Time (in seconds) registered for different behaviors during the social interaction test in Experiment 1.

	Control	Linalool	*L. angustifolia* EO
Non-social exploration	534 ± 5	494 ± 9 **	491 ± 12 **
Exploration from a distance	3 ± 0.3	2 ± 0.3	2 ± 0.4
Social investigation	50 ± 6	83 ± 8 *	84 ± 10 *
Unit of social investigation	1 ± 0.2	3 ± 0.2 ***	2 ± 0.3 **
Latency of social investigation	20 ± 3	13 ± 2	12 ± 2

Control (saline) (*n* = 15), Linalool (100 mg/kg) (*n* = 15) *L. angustifolia* EO (200 mg/kg) (*n* = 15). Results are presented as mean values ±SEM. * *p* < 0.05, ** *p* < 0.01, *** *p* < 0.001, differences with the control groups.

**Table 2:** Effects of linalool and LA essential oil on socially stressed mice in the EPM.

	Control (Explora)	SD Saline	SD Linalool	SD *L. angustifolia* EO
**Time in open arms**	52 ± 7	27 ± 4 *	21 ± 6 **	34 ± 7
**% Time in open arms**	22 ± 2	13 ± 3	10 ± 3 *	14 ± 4
**Time in central platform**	38 ± 4	50 ± 9	37 ± 12	40 ± 10
**Time in closed arms**	169 ± 14	213 ± 13	244 ± 18 **	225 ± 16 *
**Entries in open arms**	24 ± 4	26 ± 6	9 ± 3 *	15 ± 2
**% Open entries**	32 ± 3	35 ± 5	29 ± 7	31 ± 4
**Entries in closed arms**	49 ± 5	42 ± 3	22 ± 6 ***	33 ± 4
**Total entries**	73 ± 7	68 ± 8	28 ± 8 ***	48 ± 6 *

Results are presented as mean values ±SEM. * *p* < 0.05; ** *p* < 0.01; *** *p* < 0.001 differences with the control (Explora) group.

**Table 3:** Effects of linalool and LA essential oil on socially stressed mice in the different behavior parameters of the social interaction test.

	Control (Explora)	SD Saline	SD Linalool	SD *L. angustifolia* EO
**Non-social exploration**	448 ± 14	492 ± 8 *	433 ± 13 ^##^	447 ± 12 ^#^
**Exploration from a distance**	3 ± 0.5	3 ± 1	3 ± 0.4	3 ± 0.4
**Social investigation**	121 ± 12	77 ± 6 **	132 ± 9 ^##^	129 ± 12 ^##^
**Unit of social investigation**	4 ± 1	3 ± 0.1	4 ± 0.4	4 ± 0.4
**Latency of social investigation**	12 ± 3	17 ± 3	15 ± 2	15 ± 2

Time (seconds) registered for each behavior of Control (Explora) (*n* = 11); SD Saline (*n* = 10); SD Linalool (*n* = 12) and SD *L. angustifolia* EO (*n* = 12). * *p* < 0.05; ** *p* < 0.01 differences with the Control (Explora) group; ^#^ *p* < 0.05; ^##^ *p* < 0.01 differences with SD Saline.

**Table 4:** Ethological analyses of the social defeat.

Social Defeat	Control (Exploration)	Acute Linalool	Chronic *Linalool*	Acute *L. angustifolia* EO	Chronic *L. angustifolia EO*
**Intruder mice**					
**Avoidance**	82 ± 9	66 ± 6	57 ± 9	57 ± 13	37 ± 4 **
**Latency avoidance**	12 ± 5	6 ± 2	7 ± 2	19 ± 9	16 ± 4
**Defense/Submissive**	48 ± 9	51 ± 11	43 ± 5	34 ± 12	10 ± 4 *
**Latency Defense/Submissive**	27 ± 9	11 ± 3	20 ± 7	111 ± 43 *	21 ± 6
**Resident mice**					
**Threat**	3 ± 1	2 ± 0.3	3 ± 1	1 ± 0.3	22 ± 1 ****
**Latency threat**	18 ± 6	17 ± 7	53 ± 30	28 ± 9	88 ± 37
**Attack**	33 ± 5	18 ± 2 **	25 ± 3	10 ± 2 ****	6 ± 2 ****
**Latency attack**	9 ± 5	15 ± 7	5 ± 2	37 ± 26	95 ± 36 *

Results are presented as mean values ±SEM. * *p* < 0.05; ** *p* < 0.01; **** *p* < 0.0001 differences with the control (Exploration) group.

**Table 5:** Effects of linalool and *L. angustifolia* essential oil on stressed mice in the EPM.

	Control (Explora)	SD Saline	SD Acute Linalool	SD Chronic Linalool	SD Acute *L.* *angustifolia* EO	SD Chronic *L. angustifolia* EO
**Time in open arms**	57 ± 6	21 ± 3 ***	29 ± 6 *	25 ± 6 **	45 ± 7	28 ± 5 *
**% Time in open arms**	21 ± 2	8 ± 2 *	12 ± 2	12 ± 4	18 ± 4	14 ± 2
**Time in central platform**	27 ± 6	23 ± 9	39 ± 12	50 ± 13	40 ±11	74 ± 18 *
**Time in closed arms**	229 ± 11	243 ± 14	231 ± 15	237 ± 19	213 ±17	208 ± 20
**Entries in open arms**	22 ± 4	17 ± 2	16 ± 3	13 ± 3	23 ± 4	18 ± 3
**% Open entries**	36 ± 9	40 ± 4	36 ± 5	35 ± 5	43 ± 3	36 ± 4
**Entries in closed arms**	39 ± 6	31 ± 6	25 ± 5	20 ± 4 *	31 ± 4	28 ± 3
**Total entries**	61 ± 9	49 ± 7	41 ± 8	33 ± 7 *	54 ± 7	46 ± 5

Data are presented as mean values ±SEM. * *p* < 0.05; ** *p* < 0.01; *** *p* < 0.001 differences with the control group.

**Table 6:** Time (in seconds) registered for different behavior parameters in the social interaction test in stressed mice.

	Control (Exploration)	SD Saline	SD Acute Linalool	SD Chronic Linalool	SD Acute *L.* *angustifolia* EO	SD Acute *L.* *angustifolia* EO
**Non-social exploration**	454 ± 11	511 ± 8 **	447 ± 12 ^###^	459 ± 11 ^##^	469 ± 13 ^#^	446 ± 12 ^###^
**Explore from a distance**	2 ± 0.3	3 ± 0.4	2 ± 0.3	2 ± 0.3	2 ± 0.4	2 ± 1
**Social investigation**	110 ± 11	63 ± 8 **	126 ± 12 ^###^	115 ± 10 ^##^	96 ± 6	114 ± 11 ^##^
**Unit of social investigation**	4 ± 0.4	2 ± 0.2	3 ± 0.3	4 ± 1	3 ± 0.6	4 ± 0.4
**Latency of social investigation**	8 ± 2	9 ± 3	7 ± 2	20 ± 5 *^,#^	8 ± 2	15 ± 0.3

Control (Exploration) (*n* = 12), SD saline (*n* = 12); SD Linalool (*n* = 12); SD *L. angustifolia* EO (*n* = 12). * *p* < 0.05; ** *p* < 0.01 differences with the Control (exploration) group; ^#^ *p* < 0.05; ^##^ *p* < 0.01; ^###^ *p* < 0.001; differences with SD saline.
